# Monitoring osteoarthritis progression using near infrared (NIR) spectroscopy

**DOI:** 10.1038/s41598-017-11844-3

**Published:** 2017-09-13

**Authors:** Isaac O. Afara, Indira Prasadam, Zohreh Arabshahi, Yin Xiao, Adekunle Oloyede

**Affiliations:** 1 Department of Applied Physics, University of Eastern Finland, Kuopio, Finland; 2 School of Chemistry, Physics and Mechanical Engineering, Science and Engineering Faculty, Queensland University of Technology, Brisbane, Queensland Australia; 3 Institute of Health and Biomedical Innovation, Queensland University of Technology, Brisbane, Queensland Australia; 4 Australia-China Centre for Tissue Engineering and Regenerative Medicine, Queensland University of Technology, Brisbane, Queensland Australia

## Abstract

We demonstrate in this study the potential of near infrared (NIR) spectroscopy as a tool for monitoring progression of cartilage degeneration in an animal model. Osteoarthritic degeneration was artificially induced in one joint in laboratory rats, and the animals were sacrificed at four time points: 1, 2, 4, and 6 weeks (3 animals/week). NIR spectra were acquired from both (injured and intact) knees. Subsequently, the joint samples were subjected to histological evaluation and glycosaminoglycan (GAG) content analysis, to assess disease severity based on the Mankin scoring system and to determine proteoglycan loss, respectively. Multivariate spectral techniques were then employed for classification (principal component analysis and support vector machines) and prediction (partial least squares regression) of the samples’ Mankin scores and GAG content from their NIR spectra. Our results demonstrate that NIR spectroscopy is sensitive to degenerative changes in articular cartilage, and is capable of distinguishing between mild (weeks 1&2; Mankin <=2) and advanced (weeks 4&6; Mankin =>3) cartilage degeneration. In addition, the spectral data contains information that enables estimation of the tissue’s Mankin score (error = 12.6%, R^2^ = 86.2%) and GAG content (error = 7.6%, R^2^ = 95%). We conclude that NIR spectroscopy is a viable tool for assessing cartilage degeneration post-injury, such as, post-traumatic osteoarthritis.

## Introduction

Articular cartilage is a highly specialized and resilient connective tissue that functions to distribute physiological loads to the underlying bone without developing unacceptably high stresses^1^. Cartilage function can be altered by degenerative changes in its structure and composition, often as a consequence of injury. Cartilage injuries may result in acute lesions, which without intervention may progress to post-traumatic osteoarthritis (PTOA)^2^. Several techniques are available for cartilage repair, with recent research suggesting that pharmaceutical interventions may be effective in preventing the onset or halting the progression of PTOA if the injury is detected early^2, 3^. Thus, characterization of cartilage integrity and disease progression at the early disease stages is crucial for effective management and treatment of PTOA^2^.

Current clinical diagnosis of joint pathologies often involves clinical examination, with radiographic (X-ray) examination and/or magnetic resonance imaging (MRI) conducted to verify diagnosis. This is then followed by repair surgery via arthroscopic intervention. Arthroscopy enables detailed description of lesion size and severity; however, the method is ineffective in detecting early degenerative changes in cartilage. In addition, the reproducibility of arthroscopy has been reported to be poor^4, 5^ due to its subjective nature. Thus, appropriate diagnostic methods capable of detecting the onset and progression of cartilage degeneration, both objectively and in real-time, is required.

Cartilage integrity can be characterized histologically using the Mankin grading system^6^. Although this method is effective for overall tissue matrix characterization, it requires destructive (biopsy excision) and time-consuming protocols for histological evaluation of cartilage health. Direct application of this technique is therefore not feasible in surgical applications. Consequently, non-destructive approaches for determining articular cartilage health indirectly via the Mankin score have been proposed, including near infrared (NIR) spectroscopy^7–14^, mid-infrared spectroscopy^15, 16^ and optical coherence tomography (OCT)^17, 18^. This study investigates the capacity of NIR spectroscopy for detecting and characterizing progressive degenerative changes in articular cartilage.

NIR spectroscopy is a vibrational spectroscopic technique that is sensitive to specific molecular species containing CH, NH, OH and SH bonds, which constitute the fundamental chemical structure of biological tissues. NIR has been shown to be sensitive to micro- and macroscopic properties of cartilage^10, 11, 19, 20^, and a typical spectrum incorporates latent information on structural, compositional and morphological properties of the tissue. In addition, NIR spectroscopy is a rapid, non-destructive optical technique that penetrates deep into soft tissues^21^, permitting full-depth cartilage probing^22, 23^. The potential of NIR spectroscopy for non-destructive probing of articular cartilage is currently gaining research attention^7–14, 19, 24, 25^, and its capacity for evaluation of engineered cartilage has been demonstrated^26, 27^.

Earlier, we demonstrated the potential of NIR spectroscopy for estimating articular cartilage Mankin score from its spectral response, with respect to differentiating between types and severity of cartilage degeneration^7^. However, no study has assessed the capacity of this optical method for monitoring progressive degenerative changes in cartilage. In this study, we hypothesized that NIR spectroscopy is capable of detecting and characterizing degenerative changes in articular cartilage, evaluated histologically and biochemically via Mankin score and glycosaminoglycans (GAG) content analyses, respectively. Multivariate techniques for classification (principal component analysis – PCA, and support vector machines – SVM) and regression (partial least squares regression – PLSR) combined with variable selection were utilized for investigating changes/differences in the NIR spectrum associated with disease progression relative to the Mankin score and GAG content of articular cartilage.

## Methodology

### Animals

All animal experiment and protocols were approved by the Ethics Committee of Queensland University of Technology. The animal experiment and protocols were performed in accordance with relevant guidelines and regulations of the aforementioned committee. Male Wistar rats (n = 12, 8–10 weeks old) were purchased from the Animal Resource Centre (Perth, Western Australia, Australia), each animal weighing approximately 320 g. The animals were housed under conditions that included a controlled light cycle (light/dark: 12 h each) and controlled temperature (23 ± 1 °C), and were allowed to habituate themselves to the housing facilities for at least 7 days before surgeries.

### Rat OA model and sample preparation

Experimental osteoarthritis (OA) was induced in the rats by surgical removal of the medial meniscus (meniscectomy, MSX) of the right knee as described in our previous studies^7^. The left knees were left intact and used as controls (sham). The animals were euthanized at four time points: 1, 2, 4, and 6 weeks (n = 3 animals/week) post-surgery and both knee joints, injured OA and control (sham) were removed by dissection. Subsequently, NIR spectral measurements were acquired from the tibial and femoral medial and lateral condyles of each joint. The first animal in week 1 post-injury was excluded from the study as a result of experimental error, resulting in a total of 58 measurement locations (**n**_**sham**_ = 14; **n**_**w1**_ **= **8; **n**_**w2**_ = 12; **n**_**w4**_ = 12; **n**_**w6**_ = 12). Although 44 sham sample locations were available, only 14 randomly selected locations were used in order to have similar number of samples from both sham and different diseased joints. The sham samples were randomly selected to include at least one sample location per animal. Following NIR spectral acquisition, the knee joints were processed for histological staining and sulphated glycosaminoglycan (sGAG) assay analysis.

### NIR spectroscopy

Diffuse reflectance NIR spectroscopy was performed using a Bruker MPA™ FT-NIR (Fourier Transform NIR) spectrometer (Bruker Optics, Germany), with detector spanning the full NIR spectral range (4,000–12,500 cm^−1^). The spectrometer was equipped with a custom-made fibre optic probe (dia. = 5 mm, optical window = 2 mm) consisting of seven 600 µm fibres: six peripherally positioned for transmitting the NIR light, and one centrally placed for collecting the diffusely reflected light from the tissue. The spectrometer was connected to a PC running OPUS 6.5 software (Bruker Optics, Germany) for data acquisition.

In preparation for spectral measurement, each joint is firmly held in a custom-built rig as described in our previous study^7^. Prior to sample scanning, a reference spectrum was taken from a spectralon reflectance standard – *SRS-99* (Labsphere Inc., North Sutton, USA). Spectral data was then obtained over the full wavelength range at 8 cm^−¹^ resolution, with each spectrum consisting of 16 co-added scans. The location of NIR measurement was visually noted to ensure that further analyses were conducted on tissue extracted from the same region where spectral data were acquired.

### Morphological and histological characterization of OA samples

After NIR spectroscopy, the joints were fixed in 4% paraformaldehyde and decalcified in 10% EDTA over a period of 2–3 weeks. Following decalcification, cartilage sections (surface-to-bone) from the region that was subjected to NIR spectroscopic probing were carefully extracted for histological evaluation based on Mankin score. After dehydration and paraffin embedding, two serial 5 μm sagittal sections obtained at 100 μm intervals from non-weight-bearing and weight-bearing regions, were cut from the joints and stained with safranin O–fast green. For Safranin-O/Fast Green staining, the paraffin-embedded sections were counterstained with Haematoxylin before being stained with 0.02% aqueous Fast Green for 4 min (followed by 3 dips in 1% acetic acid) and then 0.1% Safranin-O for 6 min. The slides were then dehydrated and mounted with crystal mount medium. OA severity in the joints was evaluated according to the modified Mankin histological grading system^6^, and Mankin score was assigned for each sample location by three independent assessors. The Mankin score assesses structural integrity (0–6 points), cellularity (0–3 points), matrix staining (0–4 points), and tidemark integrity (0–1 points), with a maximum score of 14 points. The final score for each sample location was determined as the most severe histological change observed in multiple sections. In the case where the scores were different among the assessors, the highest Mankin score was selected. The inter-assessor agreement was assessed using the kappa (κ) coefficient, a chance-corrected estimate of agreement. κ values of 1.00–0.81 indicate excellent agreement, 0.80–0.61 substantial agreement, 0.60–0.41 moderate agreement, 0.40–0.21 fair agreement and 0.20–0.00 slight agreement^28^.

### Biochemical quantitation of GAG content

Sulphated GAG (sGAG) assay was performed based on a protocol provided in the Blyscan sulfated Glycosaminoglycan assay kit (Biocolor Life Science Assays; Labtek, West Ipswich, QLD, Australia). sGAG content was measured at a wavelength of 595 nm by a microplate spectrophotometer (Benchmark Plus, Tacoma, WA, USA). Three measurements were obtained and repeatability coefficient calculated from Bland–Altman analysis as 1.96 times the standard deviation of the differences.

### Statistical evaluation and spectral analyses

Statistical comparison of the reference histological and biochemical measurements between joints in the different groups was performed using GraphPad Prism (ver. 6, GraphPad Software Inc.). The data were expressed as mean ± SD and were compared using one-way ANOVA, a *p*-value of less than 0.05 was considered for statistical significance.

Principal component analysis (PCA), a feature extraction and classification method^29, 30^, was employed to identify inherent patterns in the spectra of samples in the different groups, and also to reduce data dimensionality to a few uncorrelated variables (principal components – PC). Scores representing the first and second PCs, which explain variations in the original spectral data, were obtained and used to characterize/group the samples. Subsequently, support vector machines (SVM), a machine learning technique based on statistical learning, was utilized to determine decision boundaries that optimally demarcate and classify any inherent classes/groups from the PCA score plot of the samples.

Multivariate prediction models for correlating the spectral data to the samples’ Mankin score and GAG content were developed using partial least squares (PLS) regression. This multivariate technique generates predictive models that optimizes the variance in both predictor variables (NIR data) and response variables (Mankin score and GAG content)^31^. The performance of the developed model is then evaluated using validation techniques, such as cross-validation. Prior to PLS regression, variable selection techniques and spectral pre-processing were applied.

Variable selection algorithms based on shaving and genetic algorithm were applied to determine the most informative spectral variables. In shaving variable selection method, a model is first fitted to the entire data, the variables are then sorted according to their selectivity ratio, a filter method for extracting a set of important variables. The selectivity ratio (SR) is defined as the ratio between explained and residual variance of the spectral variables on the target-projected component^32^. It provides a numerical assessment of the usefulness of each variable in a regression model; the larger the SR, the more useful the given variables are for the prediction. Secondly, a threshold is used to eliminate a subset of the least informative (worst performing) variables. Then a model is fitted again to the remaining variables and performance is measured. The procedure is repeated until maximum model performance is achieved^33^. Genetic algorithm is a subset search algorithm inspired by biological evolution theory and natural selection^34^. In this variable selection algorithm, an initial population of variable sets are built by random selection. A PLS model is then fitted to each variable set, and performance computed using cross-validation procedure. A collection of variable sets with higher performance are then selected to survive until the next “generation”. Subsequently, new variable sets are formed by crossover of selected variables between the surviving variable sets, and by changing (mutating) the random selection for each variable. The procedure is then repeated with the surviving and modified variable sets until optimal model performance is achieved.

Following variable selection, spectral pre-processing methods including multiplicative scatter correction (MSC) and derivative pre-treatment, as well as combinations of both methods, were applied in order to reduce spectral non-linearities resulting from light scattering variations in reflectance spectroscopy^35^ and to eliminate unwanted baseline variations, respectively. Subsequently, the resulting data (pre-processed most informative variables) are subjected to PLS analysis and then model performance is measured. The procedure is applied to both raw pre-processed spectral data.

Leave-one-out (LOO) cross-validation was employed to determine the optimal number of PLS components, and to estimate the performance of the predictive models developed. This validation method was adopted because it is effective for small sample sizes. To potentially minimise under- or over-fitting, optimal model selection was based on the lowest root mean square error of cross-validation (RMSECV), minimum number of components and high coefficient of determination (R^2^). For multivariate spectral analyses, R statistical software^36^ (ver. 3.3.1) was employed with the following packages: **stats** ^36^ for PCA analyses, **e1071** ^37^ for SVM, **pls** ^38^ for PLS analyses, and **plsVarSel** package^39^ for shaving and genetic algorithm variable selection.

## Results

### Variation of NIR spectra with tissue degeneration

**Figure 1:**
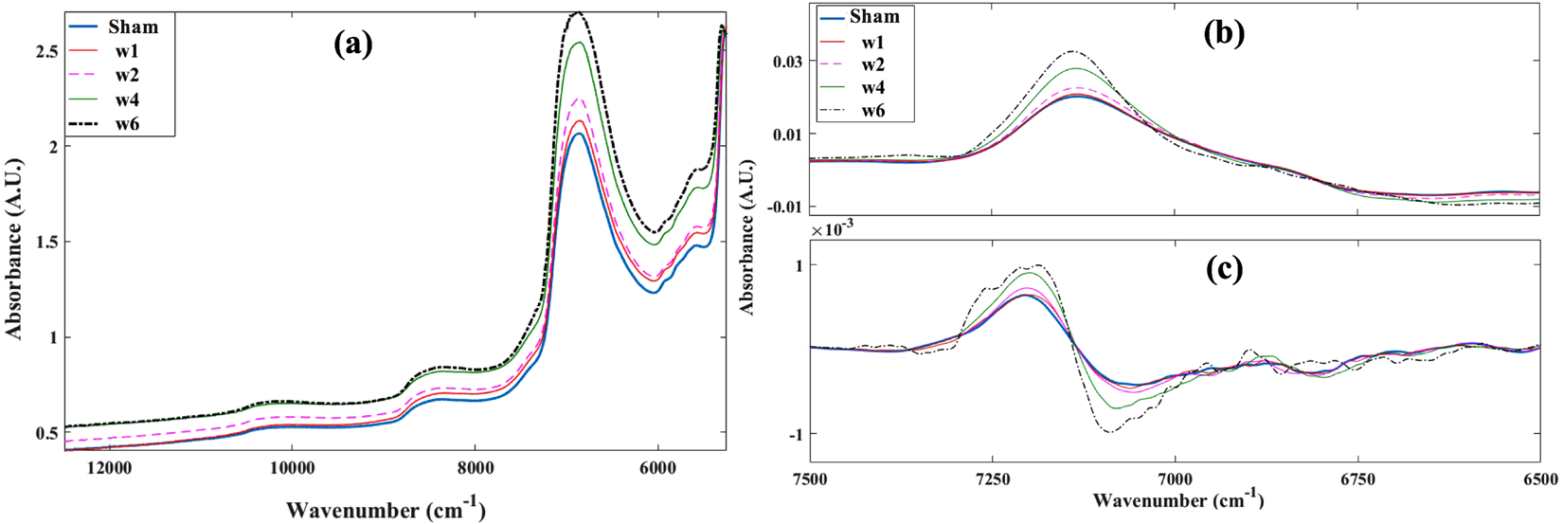
Representative raw (**a**), 1^st^ derivative (**b**) and 2^nd^ derivative (**c**) cartilage NIR spectra from control (sham) and progressively degenerating joint samples. The derivative spectra show consistent changes with progression of tissue degeneration.

**Figure 2:**
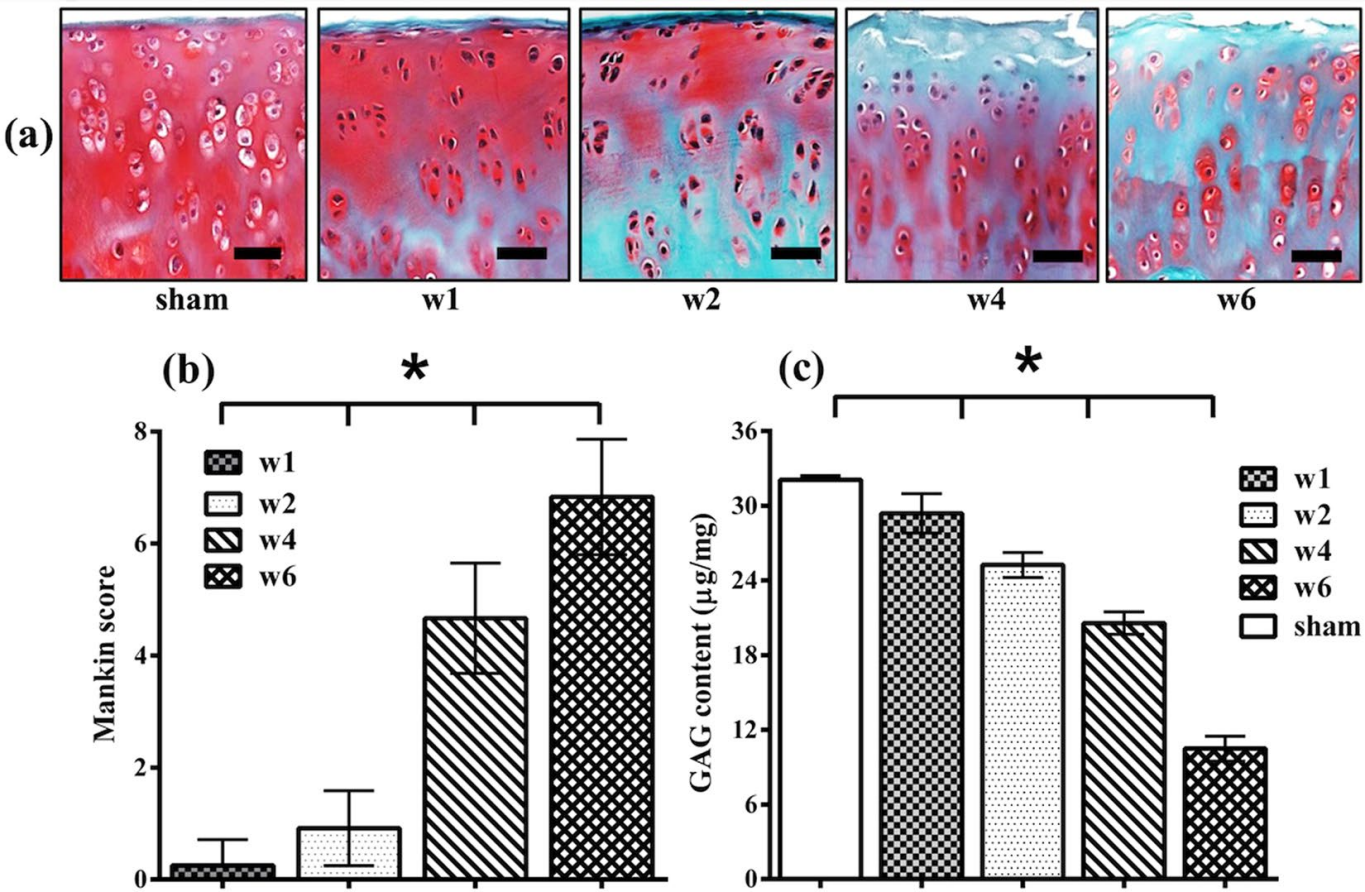
Progressive degenerative changes in an animal model of OA. **(a)** Representative histology sections of the tibial condyles for the sham (control) joint and at 1, 2, 4 and 6 weeks post-injury. [Distal tibia was sectioned coronally and stained with Safranin-O. Scale bar = 200 μm]. The most degenerated area (medial part) of each sample was selected. **(b)** Quantitation of safranin-O stained sections using the Mankin histopathology scoring system for the progressively degenerated cartilage samples. **(c)** Quantitation of the GAG content of the samples. Values are the mean ± SD; *p < 0.001.[w1 = week 1; w2 = week 2; w4 = week 4; w6 = week 6].

Variations in the optical response of the injured joints, as tissue degeneration progresses, is mostly characterized by an overall increase in absorbance across the NIR spectral range (Fig. 1). This is consistent with morphological and histological changes in the samples, which can be observed to change consistently with progression of tissue degeneration (Fig. 2). Spectral data below 5250 cm^−1^ were excluded due to spectral saturation in the 1^st^ overtone water peak and combination region of the NIR spectral range.

The severity of cartilage injury with progressive tissue degeneration, evaluated using the modified Mankin score, were observed to increase consistently (mean ± sd): 0.0 ± 0.0 for sham, 0.3 ± 0.5 for samples after 1 week (w1), 0.9 ± 0.7 2 weeks (w2), 4.7 ± 0.9 4 weeks (w4), and 6.8 ± 1.0 6 weeks (w6) post-injury (Fig. 2b). Substantial agreement was observed between the independent assessors of Mankin score (κ = 0.65). The GAG content obtained biochemically were observed to consistently decrease with progression of tissue degeneration, with repeatability coefficient of 1.24 μg/mg and the following average values: 32.1 ± 0.7 μg/mg for sham, 29.3 ± 1.4 μg/mg for w1, 25.2 ± 1.2 μg/mg for w2, 20.6 ± 1.1 μg/mg for w4, and 10.5 ± 0.9 μg/mg for w6 (Fig. 2c). This is supported by the consistent decrease in staining intensity in the corresponding histological sections of the samples due to progressive loss of proteoglycans as injury severity progresses with time (Fig. 2a).

In general, reduction in cartilage proteoglycan content, extensive alterations characterized by marked hypercellularity due to cloning, numerous osteophytes on the margins, and decrease in the cartilage thickness were observed to increase with disease progression (Fig. 2). Statistically significant difference (p < 0.001) in Mankin score and GAG content were observed between the samples at different weeks post-injury.

### Multivariate classification of tissue degeneration

Variable reduction and classification based on PCA show that the samples can be grouped into two linearly separable classes based on variations in their NIR spectral data using the 1^st^ and 2^nd^ principal components scores (PC_1_ and PC_2_). The scores can be observed to group the samples according to level of degeneration along PC_1_, while samples within each group cluster along both PC_1_ and PC_2_.

**Figure 3:**
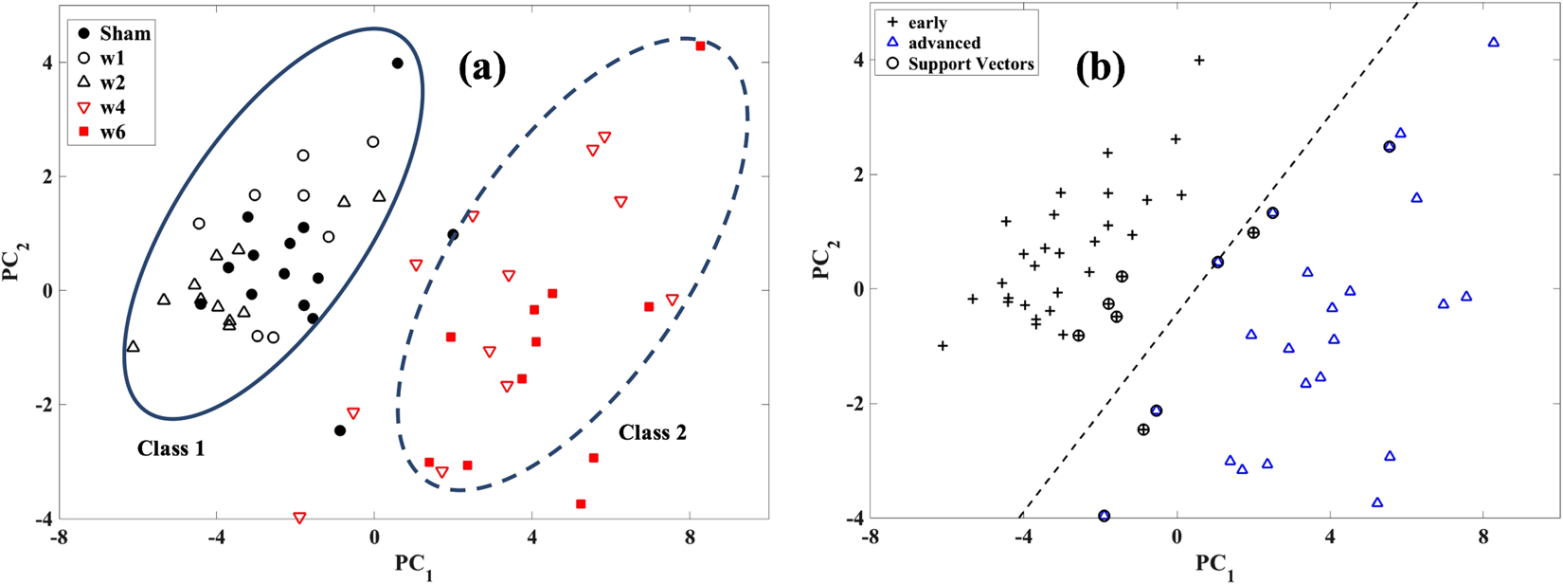
(**a**) PCA score plot of the NIR spectral data of the samples showing classification into two distinct groups. “Class 1” consists of sham and samples from weeks 1 and 2 post-injury, class 2 consists of samples from weeks 4 and 6 post-injury. (**b**) SVM decision boundary showing the optimal demarcation of both classes.[w1 = week 1; w2 = week 2; w4 = week 4; w6 = week 6].

“Class 1” consists of samples with low Mankin score (<=2) and relatively high GAG content (>23 μg/mg), and is representative of cartilage with mild degeneration (sham, weeks 1 and 2); while “class 2” consists of samples with relatively high Mankin score (=>3) and low GAG content (<23 μg/mg), indicative of advanced tissue degeneration (weeks 4 and 6) (Fig. 3). Although PCA was performed on the spectra with and without pre-processing (multiplicative scatter correction (MSC) and derivative (1^st^ and 2^nd^)), optimal classification was obtained without pre-processing. SVM shows that a decision boundary that optimally demarcates both classes in the PCA score plot can be obtained, since the classes are linearly separable (Fig. 3a). The SVM model classified all samples with advanced degeneration correctly, but misclassified two samples with mild degeneration (Fig. 3b). No significant difference (p = 0.0588) in tissue degeneration (via the Mankin score) was observed between the samples in class 1; however, statistically significant difference (p = 0.009) was observed between the samples in class 2.

### Multivariate analyses: spectral pre-processing, variable selection and PLSR

**Figure 4:**
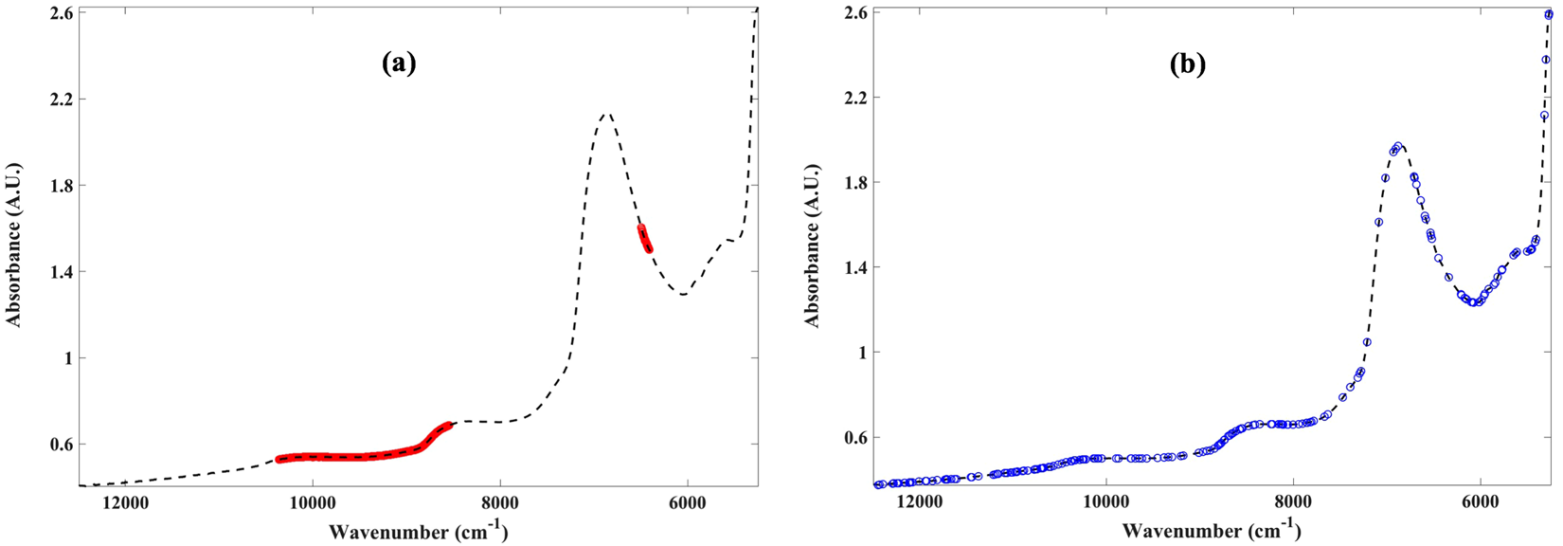
Spectral plots showing regions with the most informative variables obtained from variable selection algorithms for optimal PLS regression models for (**a**) Mankin score and (**b**) GAG content.

A combination of MSC spectral pre-processing and variable selection based on shaving resulted in the best models for estimating the Mankin score, while variable selection alone, based on genetic algorithm, was optimal for estimating the GAG content from the NIR spectra of the samples (Table 1). The most informative variables for estimating the Mankin scores from the spectral data consisted of 493 variables from two main spectral regions: 8547–10361 cm^−1^ and 6411–6496 cm^−1^ (Fig. 4a), while the model for estimating GAG content required 192 variables (Fig. 4b) broadly dispersed across the whole spectral range, with sparse variables around the 2^nd^ overtone water peak (around 6860 cm^−1^).

**Table 1:** Multivariate analyses assessment of the relationship between the optical characteristics of articular cartilage and its matrix integrity during progressive tissue degeneration.

**MANKIN SCORE**					
**Pre-pro + VS**	**n**_**comp**_	**R** ^**2**^_**cv**_	**RMSECV**	% error*	**n**_**vars**_
none	9	81.23	1.18	14.75	all
MSC	10	64.2	1.63	20.38	all
Shaving	9	87.64	0.95	11.92	1503
MSC + Shaving	8	86.22	1.01	12.60	493
GA	10	80.20	1.18	14.81	211
MSC + GA	10	85.40	1.04	13.01	201
**GAG CONTENT**					
**Pre-pro + VS**	**n**_**comp**_	**R** ^**2**^_**cv**_	**RMSECV (µg/mg)**	% error*	**n**_**vars**_
none	9	95.17	1.71	7.46	all
MSC	10	61.57	4.81	20.94	all
Shaving	8	94.47	1.83	7.97	616
MSC + Shaving	8	87.17	2.78	12.10	493
GA	9	95	1.74	7.57	192
MSC + GA	10	69.75	4.26	18.53	204

[Pre-pro = spectral pre-processing; VS = variable selection; n_comp_ = number of PLS components; RMSECV = root mean square error of cross-validation; n_vars_ = number of spectral variables used in the multivariate analyses. *Error is estimated relative to the range of the reference data, all = 1879 variables].

**Figure 5:**
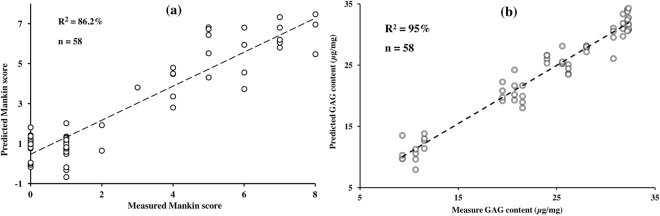
Relationship between NIR spectral predicted and measured (**a**) Mankin score and (**b**) GAG content of the progressively degenerated cartilage samples.

High correlation with relatively low error were obtained with the optimal models for Mankin score (R^2^ = 86.2%, error = 12.6%) and GAG content (R^2^ = 95%, error = 7.6%). The relationship between the measured and NIR predicted Mankin scores and GAG contents are presented in Fig. 5. The significantly high correlation between the optical response and both the tissue’s structural integrity (Mankin score) and composition (GAG content) demonstrates the capacity of NIR to monitor progression of cartilage degeneration, and ultimately OA progression.

## Discussion

In this study, we demonstrate the capacity of NIR spectroscopy to monitor changes in articular cartilage matrix during degeneration. In earlier studies^7, 9^, we demonstrated the potential of NIR to identify, classify and predict different types and severity of cartilage degeneration using rat models of OA. In these studies, only one time-point (8 weeks post-injury) was considered. However, in the present study 4 time-points (1, 2, 4 and 6 weeks post-injury) were considered, with assessment of overall tissue integrity (Mankin score) and GAG contents, thus extending the approach to monitor progression of tissue degeneration based on the hypothesis that NIR spectroscopy is capable of detecting and characterizing histological and biochemical changes in cartilage during degeneration. The results show that this optical technique is capable of classifying the samples based on severity of degeneration, as well as predict their Mankin scores and GAG contents with relatively low error.

Since absorptions in the NIR spectral range arise mainly from OH, CH, NH and SH bonds, which characterize the fundamental molecular make-up of biological materials, NIR spectroscopy is capable of detecting both micro- and macroscopic changes in articular cartilage structure and composition^10, 11, 14, 19, 20^. Thus, the capacity of NIR to effectively monitor cartilage degeneration can be attributed to the characteristics of the spectrum, which embeds information on key chemical^19^, physical^11, 22^ and morphological properties of the tissue^7, 9^. The overall increase in absorption across the spectrum as cartilage degeneration progresses (Fig. 1) is due to increase in the matrix water content, which is an expected consequence of degenerative changes in cartilage matrix composition and structure (Fig. 2), and is among the earliest indicators of cartilage degeneration.

Classification based on PCA provides an insight into the relationship between the optical response of articular cartilage and the state of its matrix, and suggests that there are subtle spectral similarities between the samples, giving rise to the two classes that cluster according to level of tissue degeneration. Separation between the classes occurs along the 1^st^ PC axis (Fig. 3a), indicating that this axis is associated with tissue integrity. Although there is clear separation between both classes, no obvious grouping can be observed within class 1, suggesting that the effect of degenerative changes on the optical response of cartilage within the first two weeks post-injury is not substantial. In addition, no statistically significant difference (p = 0.0588) in the Mankin score of the samples in this class was observed, possibly due to very early sample-specific matrix changes. However, this was not the case for class 2, where statistically significant difference (p < 0.001) was observed between samples from week 4 and week 6. A potential trend in grouping within this class can be observed, albeit with some overlap (Fig. 3a). The SVM decision boundary shows the region in space where the PC scores of new samples is likely to be located following PCA analysis. This could be an important preliminary diagnostic check to classify a sample prior to further diagnostics.

The optimal predictive model for estimating Mankin score from the NIR spectra in this study was obtained with a combination of MSC spectral pre-processing and variable selection based on shaving (Table 1). This is different from our previous study^7^ where the relationship was optimized with a combination of SNV and 1^st^ derivative pre-processing. This is probably due to the statistical variable selection technique employed in the present study, as opposed to the manual approach adopted in our previous study. In addition, MSC enables separation of tissue light absorption from physical light scattering, and has been shown to correct the undesirable effect of light scatter in NIR diffuse reflectance spectroscopy^35^. The coefficient of determination (R^2^) obtained after cross-validation is similar in both studies, with slightly higher error in the present study.

The most informative spectral variables for predicting the Mankin score are confined to two main spectral regions: 8547–10361 cm^−1^ and 6411–6496 cm^−1^ (Fig. 4a), which are indicative of the solid extracellular matrix components of cartilage. The first region (8547–10361 cm^−1^) is associated with the 2^nd^ overtone –CH2 stretch vibration, which is due to the combined proteoglycan and collagen spectral absorption. The second region (6411–6496 cm^−1^) can be attributed to proteoglycan-related spectral absorptions associated with 1^st^ overtone –NH stretch vibrations. It is worth noting that although the water content is consistently changing with tissue degeneration, spectral data associated with the main (1^st^ overtone) OH absorption region (6700–7200 cm^−1^) and directly related to cartilage water content were not optimal for predicting the tissue’s Mankin score. However, the interaction between the tissue’s water content and its changing solid matrix components is the likely driver of the relationship obtained with data from the variable selected regions.

The variables selected based on genetic algorithm for estimation of cartilage GAG content are dispersed across the whole spectrum (Fig. 4b), arguably due to the close relationship between the proteoglycan and water contents of cartilage, as the latter can be observed to increase consistently with degeneration and affects overall spectral absorption (Fig. 1). Only very few variables were required around the water peak (Fig. 4b), suggesting that data within this region does not add much information to the predictive model. The model based on the whole spectrum (without variable selection: 1879 variables) is similar to that obtained with genetic algorithm (variable selection: 192 variables) in terms of coefficient of determination and error. This is due to the dispersive nature of the genetic algorithm selected variables, which spread across the whole spectrum. However, the model based on genetic algorithm is preferable over that based on the full spectrum, since only about 10% of the variables is required to estimate the GAG content, resulting in a simpler and more robust model.

The strong correlation (Fig. 5) between the measured and predicted tissue properties (Mankin score and GAG content) demonstrates the potential of NIR spectroscopy for detecting and monitoring cartilage degeneration, as well as a tool for clinical diagnosis of cartilage condition post-injury during surgery. However, clinical adaptation of the outcome of this study would need to be preceded by extensive and rigorous pre-clinical validation and optimization using human tissue samples.

This study contributes to knowledge that could advance understanding of the degenerative process of cartilage post-injury, as well as potential clinical monitoring of the extent of cartilage degeneration during surgery, particularly in relation to PTOA, thus enabling more focused and optimized treatment regiment. Application of variable selection techniques, which significantly reduces the number of spectral data required for prediction of cartilage properties, presents a computationally faster approach compared to using the whole spectrum. This translates to less time for analyses and better support for real-time application, such as mapping tissue properties around lesions during surgery. In addition, the outcome of this study presents potential applications beyond cartilage degeneration, to monitoring the progression of other soft tissue pathologies.

Although the Mankin scoring system is not widely used clinically for assessing tissue degeneration, it provides detailed histological information on the tissue health. Nevertheless, the analytical techniques applied in this study can be extended to clinically applicable scoring systems, such as OARSI system. Another limitation of the current study is that tissue degeneration in humans may not follow the exact pattern observed in the rat samples, as osteoarthritic degeneration tend to be complex in nature. However, the injury model employed, which was surgically initiated, is effective in modeling the characteristics of natural OA^40^. Finally, the present study did not evaluate the capacity of NIR spectroscopy to estimate collagen-related information, such as cross-linking and fibril orientation, which are not accounted for in the Mankin scoring system.
